# Patterns of referral for refugees in western Tanzania: a retrospective review

**DOI:** 10.11604/pamj.2022.41.76.32559

**Published:** 2022-01-27

**Authors:** Orit Abrahim, Sarah Rapaport, Hilary Ngude, Mohamed Abbas, Peter J Winch, Kent A Stevens, Zachary Obinna Enumah

**Affiliations:** 1 Johns Hopkins Global Surgery Initiative (JHGSI), Department of Surgery, Johns Hopkins Hospital, Baltimore, MD, USA,; 2 Tanzania Red Cross Society, Dar es Salaam, Tanzania,; 3 Department of International Health, Johns Hopkins School of Public Health, Baltimore, MD, USA

**Keywords:** Refugee health, referral, Tanzania, surgery

## Abstract

**Introduction:**

access to essential secondary and tertiary healthcare, including surgery and medical sub-specialties, is a challenge in low-and-middle income countries (LMICs), especially for displaced populations. Referrals from refugee camps are highly regulated and may pose barriers to accessing essential secondary healthcare in a timely manner. Refugee referral systems and the ways they interact with national systems are poorly understood. Such information is necessary for resource allocation and prioritization, optimizing patient outcomes, national-level planning, and investment in capacity-building.

**Methods:**

a retrospective review of referrals from Nyarugusu Refugee Camp in Tanzania to Kabanga Hospital between January 2016-May 2017 was conducted. Data was collected from logbooks on patient demographics, diagnosis, and reason for referral. Diagnoses and reasons for referral were further coded by organ system and specific referral codes, respectively.

**Results:**

there were 751 entries in the referral logbook between January 2016 and May 2017. Of these, 79 (10.5%) were excluded as they were caretakers or missing both diagnoses and reason for referral resulting in 672 (89.5%) total entries for analysis.The most common organ system of diagnosis was musculoskeletal (171, 25.5%) followed by head, ear, eye, nose and throat (n=164, 24.4%) and infectious disease (n=92, 13.7%). The most common reason for referral was imaging (n=250, 37.2%) followed by need for a specialist (n=214, 31.9%) and further management (n=116, 17.3%). X-ray comprised the majority of imaging referred (n=249, 99.6%). The most common specialties referred to were ophthalmology (n=104, 48.6%) followed by surgery (n=63, 29.4%), and otolaryngology (ENT) (n=17, 7.9%).

**Conclusion:**

given a large burden of referral for refugee patients and sharing of in and out-of-camp healthcare facilities with nationals, refugees should be included in national health care plans and have clear referral processes. Epidemiological data that include these intertwined referral patterns are necessary to promote efficient resource allocation, reduce unnecessary referrals, and prevent delays in care that could affect patient outcomes. International agencies, NGOs, and governments should conduct cost analyses to explore innovative capacity-building projects for secondary care in camp-based facilities.

## Introduction

Access to essential secondary and tertiary referral healthcare, including surgery and medical sub-specialties, is a challenge in low-and-middle-income countries (LMICs) [1]. For displaced populations (e.g. migrants, refugees, internally displaced persons) residing in LMIC host countries, access to healthcare is particularly complex and is further dictated by policies and practices created in partnership with governments, the United Nations High Commissioner for Refugees (UNHCR) and non-governmental organizations (NGOs). In 2009, UNHCR´s Principles and Guidance for Referral Health care for Refugees and Other Persons of Concern provided an initial overarching framework for access to referral care, which has since evolved into advocacy to integrate displaced populations into national health systems [2,3]. Although many countries have begun to include refugees in national health plans, health screening, established referral protocols, health financing, and access to health facilities are still limited for refugees. Language, limited health education on chronic disease prevention and screening, cost, and discriminatory practices are a few of the barriers refugees face in accessing timely, culturally-sensitive, and affordable primary healthcare, and subsequently adequate referral [4].

In many refugee camp settings, movement outside of camps including for medical referrals is highly regulated. When the need for medical equipment, diagnosis, and treatment extends beyond the limited capabilities of camp facilities, a referral is a cornerstone of management and requires coordination with local health systems and international agencies. In Jordan, for example, NGOs work with UNHCR and a third party agency to facilitate emergency obstetric care in hospitals outside of refugee camps [5]. Efficient, well-planned referral processes were found to provide relatively timely care. Conversely, referral outside of camps can also lead to lapses in the continuity of care and the inability to track patient outcomes, as health information may not transfer between national and camp systems. The collection and integration of refugee health data into national health management information systems (HMIS), including matters related to referral care, are vital to planning inclusive national health systems [6].

Emergency care, such as for obstetrics and trauma,is commonly recognized in the development of referral systems in these settings [7]. Secondary care for chronic diseases, essential surgery, and other speciality care remains context-dependent and is less likely to be covered by current UNHCR related funding mechanisms. In Lebanon, NGOs were able to integrate chronic disease care into primary services through task-shifting and procurement of medical equipment, but non-emergent disease complications were not included in referral models due to resource constraints [8]. Much is unknown about how refugees access out-of-camp, non-emergent secondary care and for what services. As medical imaging such as point-of-care ultrasound is introduced into refugee settings to lessen the need for difficult referrals, it is important to analyze the subsequent effects on referral patterns, cost, and patient outcomes for future investments in camp-based providers, equipment, and therapies [9].

Thus, understanding the epidemiology of referral for refugees is important for resource allocation and optimizing the efficiency of health systems. Particularly for refugees residing in camps, data on which health services refugees access outside of the camp, the ways in which they access this care, and the barriers to access are poorly documented and understood. While some research does exist on referral in general for surgical and medical conditions in Tanzania in other LMICs, the literature on how refugees access referral health care is lacking [10,11]. Similarly, refugee referral patterns and access to healthcare can also aid in the prioritization of certain equipment and services to facilities based on the needs of the targeted population. The purpose of this study was to describe the referral patterns for refugees to a single district hospital in Kasulu, Western Tanzania.

## Methods

We retrospectively reviewed paper logbooks on referrals to Kabanga Referral Hospital for refugees residing in Nyarugusu refugee camp.

**Setting:** in the Kasulu District of Tanzania, the Nyarugusu Refugee Camp provides health services in multiple health facilities for over 130,000 refugees and the surrounding community. In July 2018, the population was approximately 150,000 [12]. There is one dispensary that houses inpatient wards, an outpatient centre, pharmacy and operating theatres. Additionally, there are satellite health centres and posts within the confines of the refugee camp. Patients whose care exceeds the capacity of the camp facilities are referred to district hospitals in Kasulu, which include both public and private entities, as well as other hospitals in the region, such as Maweni Regional Referral Hospital or Bugando Medical Center. The camp health system emphasizes the availability and accessibility of primary healthcare by providing camp-based services free of charge and through numerous satellite facilities, and the facilities see on average over 20,000 outpatients per month [13]. Reproductive health, nutrition, and infectious disease predominate the services provided at this level. The study site was chosen because Tanzania has a long history of hosting refugee populations for six decades. The corresponding author (ZOE) has a longstanding relationship with the humanitarian organization that runs the main medical mission in the camp. Importantly, Tanzania has a forced encampment policy, which requires them to have a permit from the Ministry of Home Affairs to leave the refugee camp for health care. While even referral services are provided for free, this creates a unique problem-space for refugees seeking referral health care.

**Data collection:** photos of the logbook recording referrals were taken and digitized into a standardized form using Microsoft Excel. The data included name (which was de-identified), date, sex, age, address of the patient, patient or caretaker status, diagnosis, and reason for referral. Data in the logbook included referrals to Kabanga Hospital between January 2016 and May 2017. Diagnoses were coded by organ system (e.g. neurological, cardiovascular, infectious disease (ID), musculoskeletal (MSK), neoplastic, head, ears, eyes, neck, throat (HEENT) etc.) and reasons for referral were coded as dental, further management, imaging, specialist, or missing. Each referral had up to two separate diagnoses and up to 3 diagnoses codes for diagnoses that involved multiple organ systems (e.g. tuberculosis was coded as ID and pulmonary). That is, a single observation or referral could be coded to multiple organ systems or reasons for referral. The reasons for referral coded as “specialist” were further coded as either surgery, ophthalmology, gynecology, otolaryngology, or orthopedics.

**Data analysis:** entries were also excluded if both the diagnosis and reason for referral fields were missing/illegible or the status indicated was caretaker (not patient). All missing values were assumed to be missing at random. A second coder analyzed approximately 10% of the data to confirm accuracy of diagnostic code and reasons for referral code assignments. Data were analyzed descriptively using Microsoft Excel and SAS software (Copyright 2012-2018, SAS SAS Institute Inc., Cary, NC, USA). Figures were also produced using Stata (Statistical Software: Release 16. College Station, TX: StataCorp, LP).

**Ethics approval:** ethical approval was obtained from the Johns Hopkins Institutional Review Board (#12663) and Tanzanian Commission on Science and Technology (2020-391-NA-2011-143).

**Ethics approval and consent to participate:** the study was carried out in accordance with relevant guidelines and regulations. Informed consent was waived by the Johns Hopkins Institutional Review Board. Study approval was also obtained from the Tanzanian Commission on Technology and Science. Consent for publication - Not applicable.

**Availability of data and materials:** the datasets generated and/or analyzed during the current study are not publicly available due to privacy reasons. De-identified, aggregate data are available from the corresponding author on reasonable request.

## Results

### Characteristics of the study and study population

**Figure 1:**
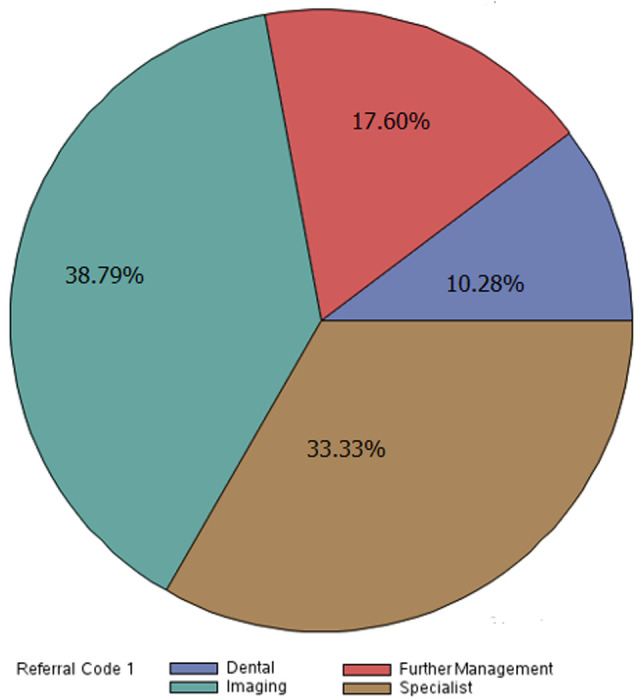
distribution of reasons for referral

There were 751 entries in the referral logbook between January 2016 and May 2017. Of these, 79 (10.5%) were excluded as they were caretakers or missing both diagnoses and reasons for referral resulting in 672 (89.5%) total entries for analysis. The median age of referred patients was 29.0 (IQR30) and 356 (53.1%, one missing) referred patients were female. Forty-one (6.1%) entries were missing only a diagnosis and 30 (4.5%) were missing only a reason for referral. One entry referred for “to control pelvis” did not specify a diagnosis and could not be given a referral code. One entry with a dilated cardiomyopathy diagnosis had a reason for referral as “dental” and was coded as such to maintain objectivity. One-hundred and twenty-eight (19%) entries received double-coded diagnoses and 9 (1.3%) received three diagnoses codes. Four entries were double-coded for reason for referral. The most common organ system of diagnosis was MSK (171, 25.5%) followed by HEENT (164, 24.4%) and ID (92, 13.7%). The most common reason for referral was imaging (n=250, 37.2%) followed by need for a specialist (n=214, 31.9%) and further management (n=116, 17.3%) (Table 1, Figure 1).

**Table 1:** description of total referrals to Kabanga Hospital

Variable	n (%), median (IQR)	Total (N)
**Age**	29.0 (30)	-
**Sex**		
Male	315 (46.9)	672
Female	356 (53.0)	
**Diagnosis**		672
Musculoskeletal	171 (25.5)	
HEENT	164 (24.4)	
Dental	67 (10.0)	
Infectious disease	92 (13.7)	
Missing	41 (6.1)	
Gastrointestinal	43 (6.4)	
Cardiovascular	40 (6.0)	
Gynecologic	34 (5.1)	
Pulmonary	47 (7.0)	
Unspecified	30 (4.5)	
Neurologic	24 (3.6)	
Soft tissue mass	12 (1.8)	
Neoplasm	14 (2.1)	
Urology	12 (1.8)	
Hematologic	8 (1.2)	
Unspecified neoplasm	5 (0.7)	
Renal	4 (0.6)	
Endocrine	6 (0.9)	
**Reason for referral**		
Imaging	250 (37.2)	
Specialist	214 (31.9)	
Further management	116 (17.3)	
Dental	66 (9.8)	
Missing	30 (4.5)	
**Type of imaging**		250
Ultrasound	1 (0.4)	
X-ray	249 (99.6)	
**Type of specialist**		214
ENT	17 (7.9)	
PT	1 (0.5)	
Gynecologist	13 (6.1)	
Ophthalmologist	104 (48.6)	
Orthopedics	16 (7.5)	
Surgeon	63 (29.4)	

### Referrals by organ system

**Figure 2:**
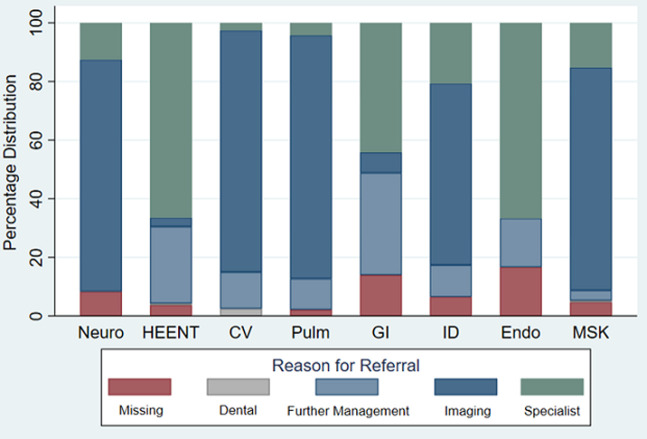
percent distribution of reason for referral by diagnostic category *Neuro= Neurology; HEENT= Head, Eyes, Ears, Nose, Throat; CV= Cardiovascular; Pulm= Pulmonary; GI= Gastrointestinal; ID= Infectious Disease; Endo=Endocrine; MSK= Musculoskeletal

**Figure 3:**
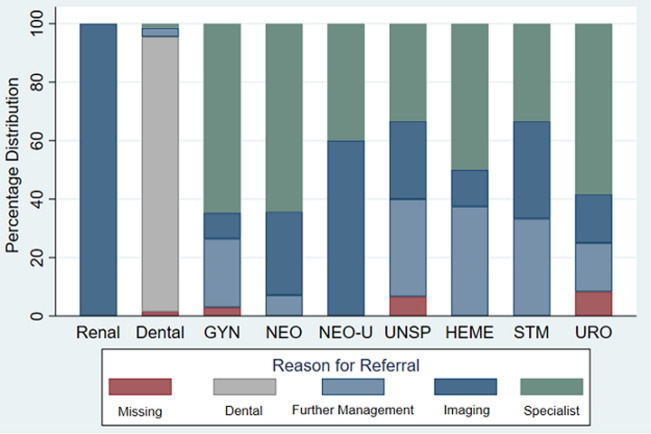
percent distribution of reason for referral by diagnostic category (Con't) *GYN= Gynecologic; NEO= Neoplasm; NEO-U= Neoplasm-Unspecified; UNSP= Unspecified; HEME= Hematology; STM= Soft Tissue Mass; Uro= Urology

The top three organ systems referred were MSK, HEENT, and ID (as above). Most MSK referrals (130, 76.0%) were for imaging, followed by specialists (26, 15.2%) (Figure 2 and Figure 3). For those referred for HEENT diagnoses, most (109, 66.5%) were referred for a specialist or further management (43, 26.2%). Endocrine, renal, and unspecified neoplasms were the least referred diagnostic codes (4, 0.6%; 5, 0.7%; 6, 0.9% respectively).

### Imaging referrals

X-ray comprised the majority of imaging referred (249, 99.6%).The most common diagnoses referred for imaging were MSK (130, 52.2%), ID (57, 22.9%), and pulmonary (38, 15.3%). Among MSK cases referred for X-ray the most common diagnoses were fracture (45, 34.6%) and osteomyelitis (16, 12.3%). Of the X-ray referrals related to ID, most (28, 49.1%) were related to tuberculosis (pulmonary, spine, meningitis) followed by osteomyelitis (15, 26.3%). The most common pulmonary X-ray referral was for pulmonary TB (26, 66.7%).

### Specialist referrals

The most common specialties referred to were ophthalmology (104, 48.6%) followed by surgery (63, 29.4%), and otolaryngology (ENT) (17, 7.9%).Of the referrals to ophthalmology, the most common diagnoses were for vision problems (45, 43.3%) which included “refractive error”, “hyperopia”, “presbyopia”, “metropia”, and “low vision”; followed by “cataract” (22, 21.2%). Of the referrals to surgery, the most common diagnosis was for intestinal obstruction (9, 14.3%). Of the referrals to ENT, the most common diagnoses were equally for nasal polyps and otitis media (3, 15.8%).

### Further management referrals

The most common referrals (excluding missing diagnoses) for further management were for HEENT (43, 37.1%), gastrointestinal (15, 12.9%), and ID (10, 8.6%). Among HEENT referrals, most diagnoses were ophthalmological (cataract 37.2%, vision problems 30.3%, optic atrophy 9.3%). Hepatic diagnoses and peritonitis were the most common gastrointestinal referral for further management (cirrhosis, liver cyst, liver disease not otherwise specified, 3, 20.0%) followed by intestinal obstruction (2, 13.3%). Most ID referrals were for pelvic inflammatory disease and TB (3, 30.0%).

## Discussion

This study describes the referral pattern from a large refugee camp in western Tanzania to a district-level hospital including the demographic profile of referred patients, the types of diagnoses referred, and the reasons for referral. Our study population had an average age of 29 years which was consistent with general camp demographics.

### Referral burden

Over a 15-month period, over 650 referrals were made to a single district hospital in the region. Importantly, this number of referrals excludes those to other hospitals that patients are often transferred to, including Maweni Regional Referral Hospital, Bugando Medical Center, and Heri Mission Hospital thus representing only a fraction of referrals to out-of-camp facilities. While referral appears to be common, data on overall referral rates, time to referral, referral completion, barriers, and outcomes is lacking. A study in 2011 of dental referrals made to Kabanga from Nyarugusu and Mtabila camps, found that treatment was cost-prohibitive to most refugees and only 50% of referred cases were treated over an almost 2 year period [14]. Without the establishment of referral processes that allow for tracking follow-up and patient outcomes, the efficiency and safety of referrals cannot be fully assessed. The push for integration of refugees into national health systems must also include plans for inclusion in health information systems and non-coercive, ethical means of monitoring refugee healthcare access and outcomes. Additionally, our results must be understood in the context of services offered in the camp. Major and minor operations are offered in the camp (e.g., caesarean section, bilateral tubal ligation, hernia repair, dilation and curettage), but diagnostic equipment and more invasive procedures (e.g., laparotomy for bowel obstruction) are often unavailable. We do not suggest our results should be taken as a determination of the underlying need or full caseload in the camp. Importantly, though, services for refugees in the camp and any referrals made outside the camp are free of charge for refugees.

### Referral for chronic diseases

This analysis also found that chronic disease and cancer contribute to the burden of disease faced by this population. These included cases such as dilated cardiomyopathy, rheumatic heart disease, chronic bronchitis, and cancer of the cervix, one of the most common types of cancers in Tanzania [15]. Refugees may stay in exile for up to 58 years meaning that for many refugees with complex chronic diseases, repeated and longitudinal follow-up is required after the initial referral and for decades after [16]. In Nyarugusu, follow-up visits for chronic conditions require regular re-evaluation for referral approval, leaving many without the guarantee that they will receive necessary longitudinal management in the camp [17]. Some barriers to access to continuous complex care in humanitarian settings have been addressed through screening programs, task-shifting, and integration into national health systems [18-20]. In some settings, the implementation of specific training programs or telemedicine consultations may lessen the need for referral without requiring additional health providers [14,20]. With innovations in service delivery, care must be taken that refugees receive equitable standards of care. This emphasizes the need for reliable, consistent data collection and health outcome monitoring.

### Preventing unnecessary referrals: a case for investment

Jumbam *et al*. investigated referrals in northern Tanzania and determined up to 35% of referrals were preventable based on national plans outlining which services are appropriate for each facility level [11]. In Nyarugusu, local health services must be fully exhausted before elective cases are considered for referral [17]. Then, the Medical Referral Committee decides whether to approve elective referrals based primarily on prognosis, followed by cost, similar to other refugee settings studied [21]. Results from our study find that certain diagnoses (e.g. uncomplicated dental caries) could be managed at the primary care level but in fact, are being referred. As others have argued, an investment in human and equipment resources will be necessary at various levels of care to both reduce unnecessary referrals and strengthen the underlying and intertwined health system between the refugee health centre and national health care structure [11]. Thus, the development of referral processes must be context-specific, taking into account the human resources, medical equipment and devices, training, and other health system building blocks for a particular setting. Regular assessments of health service availability and readiness at each facility level are needed to make these decisions and link patients to appropriate care.

### Imaging

The introduction of ultrasound to Nyarugusu refugee camp has theoretically removed a need to refer patients for this imaging. In Nduta refugee camp in Tanzania, national staff were successfully trained on point of care ultrasound and were connected to radiologists via telemedicine for clinical guidance and image interpretation [22]. Almost 40% of the referrals made to Kabanga were for imaging and these were almost entirely for X-ray. Our analysis found many of these were for fractures, and infectious and chronic cardiac and pulmonary diagnoses which may only necessitate plain film radiography. One study found the median referral time from a refugee camp to a hospital for tuberculosis care to be 18 days, with 60% of refugees experiencing long delays [23]. This is notable, given over two-thirds of pulmonary imaging referrals to Kabanga were for suspected TB, potentially prolonging time to treatment and the likelihood of transmission. An investment in an X-ray machine, for example, at the dispensary level would not only lead to the prevention of potentially unnecessary referrals but also would likely lead to decreased delays in diagnosis. Similar telemedicine training programs exist for plain film reading in sub-Saharan Africa and specifically for rural areas that could be incorporated into camp facilities [24].

Further assessment of the available services and expertise at the camp level is needed to understand the capacity to power equipment, perform imaging, troubleshoot devices, and interpret images [25]. As the access to radiologists remains limited, telemedicine and potentially artificial intelligence software may be useful resources to make in-camp X-ray feasible and safe. Introduction of new imaging modalities must consider appropriate training curricula, duration, supervision, and competency measurements [26]. Despite the initial resource investments, implementation of sustainable systems may lessen costs in the long-term for both refugees and health systems, as well as improve patient outcomes by reducing delays in care due to referral further research is needed to determine this.

### Essential Surgery

In our analysis, of all referrals, over 30% were referrals to see surgical specialists. Tanzania's National Surgical, Obstetric and Anesthesia Plan (NSOAP), however, does not include refugees or refugee camps in its referral pathway and does not accurately represent the refugee health system and its facility-based surgical services [11,27]. For example, in the NSOAP, basic general surgeries such as appendectomies, hernia repairs, and laparotomies are provided at the district level hospital. In Nyarugusu, a forthcoming review of surgical procedures revealed that these procedures are being performed with relatively high frequency at Nyarugusu camp dispensary level hospital, although district-level hospitals are available for referral [28]. Similarly, this review found that up to almost 30% of cases were for Tanzanian nationals seeking care in camp facilities. Documentation of the referral network within the camp and how it interacts with that of the national system is lacking. Although the political recognition of refugees in Tanzania is complex, the reality is that these health systems are intertwined and refugees must access facilities out of camps and nationals may access those within. Thus, national health planning must be inclusive of refugees particularly for services such as surgery where unmet needs exist among host populations as well.

In Mae La Refugee Camp, it was found that more than 11,000 USD worth of operations referred to the district hospital could have been prevented if cases were operated on in the camp while non-acute [29]. Retrospective review of surgical referrals allowed stakeholders to determine which referrals were “preventable” and could avoid the excess cost to the patient and health system. Delaying non-urgent surgical cases for referral to secondary care can lead to excess morbidity, increase the risk of complications, and worsen patient outcomes. Increasing the capacity for surgical services in refugee camps may take many forms. As suggested in previous studies, use of visiting specialist surgeons, task-shifting to non-physician providers, or training of general surgeons in advanced procedures may reduce the need for referral, and for conditions with high prevalence may reduce cost [29]. A study in Tanzania found similar outcomes between non-physician clinicians and physicians performing non-obstetric surgeries such as elective hernia repair, prostatectomy, exploratory laparotomy, and hydrocelectomy [30]. Shifting of secondary care to refugee camps and to non-traditional providers must be of equitable standards and with patient safety at the forefront. In addition to collecting epidemiological data on the burden of surgical disease, information on specific indications for referral, post-operative complications, and survival are needed.

**Limitations:** there are limitations to this study. The data represents referral to a single district hospital and may not be representative of all referrals from the camp. The types of referrals made may reflect the services provided at Kabanga and do not reflect the overall disease burden. Thus, corroborative research, including on overall referral rates, costing, and outcomes is required for resource allocation and prioritization. As data was obtained from handwritten logbooks, translation of diagnoses and reasons for referral may vary by reviewers. This subjectivity was reduced through quality checks by a second coder.

**Funding:** project supported by American Society of Tropical Medicine and Hygiene Benjamin Keane Fellowship and author ZOE received support from Ruth L. Kirschstein National Research Service Award (NRSA) T32 Appointment (Award: 2T32AR67708-6, PI: Clemens, T.) and Association for Academic Surgery Global Surgery Research Fellowship.

## Conclusion

Given a large burden of referral for refugee patients and the sharing of in and out-of-camp healthcare facilities with nationals, refugees should be included in national health care plans and have clear referral processes. Epidemiological data that includes referral patterns are necessary to promote efficient resource allocation, reduce unnecessary referrals, and prevent delays in care that could affect patient outcomes. Using this information, international agencies, NGOs, and governments should conduct costing analyses to explore innovative capacity-building solutions and the integration of certain secondary care into camp-based facilities as feasible. Referral system planning should remain equitable, allow freedom to seek referral care if desired and be patient-centred.

### What is known about this topic

- *Referral is a complex process for refugees in Tanzania;*
- *Overburdening of higher level hospitals remains a problem in Tanzania with many referrals potentially preventable*.

### What this study adds

- *To our knowledge, our study provides the first empirical analysis of refugee utilization of national health care services in Tanzania for refugees in Tanzania who access host country health services, a significant proportion may be for diagnostic purposes, such as obtaining X-rays;*
- *Study highlights the need for more robust registries tracking referrals for indications and outcomes*.
